# Rational Design and Numerical Analysis of a Hybrid Floating cIDE Separator for Continuous Dielectrophoretic Separation of Microparticles at High Throughput

**DOI:** 10.3390/mi13040582

**Published:** 2022-04-08

**Authors:** Yalin Li, Yan Wang, Georg R. Pesch, Michael Baune, Fei Du, Xiaomin Liu

**Affiliations:** 1 College of Chemistry and Chemical Engineering, Qingdao University, Qingdao 266071, China; liyalinzl0919@163.com; 2 Chemical Process Engineering, Faculty of Production Engineering, University of Bremen, Leobener Straße 6, 28359 Bremen, Germany; gpesch@uni-bremen.de (G.R.P.); mbaune@uni-bremen.de (M.B.); 3 Institute of Water Chemistry, Technische Universität Dresden, 01062 Dresden, Germany; fei.du@tu-dresden.de

**Keywords:** dielectrophoresis (DEP), high throughput, hybrid floating electrode, Joule heating, bio- and non-bioparticle separation

## Abstract

Dielectrophoresis (DEP) enables continuous and label-free separation of (bio)microparticles with high sensitivity and selectivity, whereas the low throughput issue greatly confines its clinical application. Herein, we report a novel design of the DEP separator embedded with cylindrical interdigitated electrodes that incorporate hybrid floating electrode layout for (bio)microparticle separation at favorable throughput. To better predict microparticle trajectory in the scaled-up DEP platform, a theoretical model based on coupling of electrostatic, fluid and temperature fields is established, in which the effects of Joule heating-induced electrothermal and buoyancy flows on particles are considered. Size-based fractionation of polystyrene microspheres and dielectric properties-based isolation of MDA-MB-231 from blood cells are numerically realized, respectively, by the proposed separator with sample throughputs up to 2.6 mL/min. Notably, the induced flows can promote DEP discrimination of heterogeneous cells. This work provides a reference on tailoring design of enlarged DEP platforms for highly efficient separation of (bio)samples at high throughput.

## 1. Introduction

Dielectrophoresis (DEP), in the early stages, was applied, for example, for the concentration of high-grade ore and for metal recovery in waste streams [1,2,3]. In the last decades, researchers discovered its application potential in biomedical fields due to its high precision, low cost, and label-free nature. At present, DEP has made outstanding contributions to the separation, enrichment and capture of bioparticles, such as cells, proteins, or DNA [4,5,6,7,8]. For instance, Chiou et al. [9] used tunnel DEP to achieve high-precision cell separation, in which high-purity monocytes were separated from whole blood. Cao et al. [10] employed insulator-based dielectrophoresis (iDEP) to enrich proteins for more sensitive immunoassays. Avijit et al. [11] adopted the uneven electric field generated by the sharp edges of single-layer graphene to apply DEP force on DNA molecules, allowing capture of DNA molecules with an efficiency close to 100%. Although high separation efficiency and accuracy have been achieved, these studies mainly focus on laboratory level analysis. Most DEP applications are in microsystems using on-chip devices at small sample throughput, being typically on the level of µL/min [12,13,14,15].

In fact, the miniaturized separation system limits the throughput and greatly confines the application of DEP in fields that require high throughput, i.e., the extraction of rare metals from electronic wastes and the separation of circulating tumor cells (CTCs) from the whole blood, in which the target particles are extremely low in quantity within a large volume of samples [16]. Therefore, the throughput issue needs to be addressed for further development of DEP towards industrial and/or (some) clinical applications. Based on this, some research groups have performed a variety of studies and achieved fruitful results [17]. Nie et al. [18] designed a dielectric separation device using 3D microelectrodes made of conducting PDMS to separate human cervical cancer (HeLa) cells from lymphocytes. Experimental results have shown that the proposed DEP separator can achieve a sample throughput of 20 µL/min. Aghaamoo et al. [19] combined deterministic lateral displacement (DLD) with DEP (named as deterministic DEP) for the continuous isolation of CTCs from peripheral blood cells. The high throughput of DLD makes up for the limitations of DEP, improving the processing capacity while ensuring a high separation efficiency between breast cancer cells and leukocytes. While the use of 3D microelectrodes and/or the integration of DEP with other technologies further improved the throughput of the DEP system and demonstrated its potential for clinical application in cell classification, the throughput has not yet reached the threshold of some clinical sample analysis, e.g., recovery of CTCs from peripheral blood cells [20].

A promising approach to improve the throughput is to scale up the characteristic dimension of DEP-based systems, which we have previously proven to be feasible [21,22,23]. For example, we successfully scaled-up the DEP system by tailoring the design of a continuous DEP separation channel embedded with an array of cylindrical interdigitated electrode (cIDE) for size-based fractionation of microparticles. We demonstrated that the sample throughput can reach to the level of mL/min [23]. However, the impact of Joule heating was not considered in our developed model and it was found that the experimental particle trajectories deviated from theoretical predictions especially for small particles.

Since the characteristic dimension of the DEP-based separator in many previous studies was mostly on the micrometer scale with very low voltages being applied for particle separation, the Joule heating effect was therefore rarely taken into account in these separation processes, except for some iDEP cases that require extremely high voltage input [15,24]. Generally, a relatively low voltage applied in a microsystem can generate a sufficient electric field to manipulate particles, and in some studies, suspension media with low conductivities are also used to reduce the Joule heating interference, so that the thermal impacts on the movement of particles can be basically ignored [25,26]. However, to scale up the characteristic length of a DEP platform for throughput enhancement, it is often required to increase the applied voltage so that the generated electric field gradient is sufficiently high in the entire operating region of the device to effectively manipulate particles. The high applied voltage in combination with conducting media make Joule heating a non-negligible factor, which causes the localized temperature gradient in the suspension, thereby causing electrothermal and buoyancy flow within the DEP system. Notably, studies have shown that in a DEP system with a small volume, the heat flow of the fluid is predominately caused by changes in the conductivity and permittivity of the suspending medium, whereas in an upscaled system (at which the characteristic length of the system rises from micron level to millimeter level), the heat flow is mainly dominated by buoyancy, that is, the induced fluid flow is mainly caused by the difference in the density of the suspension medium due to the temperature gradient [27,28]. The undesired fluid flow is known to affect the DEP manipulation of particles and the impact is difficult to predict and control, especially in continuous DEP separation systems [29]. Moreover, when processing biological samples, the structure and activity of particles can be adversely affected or even damaged when the temperature exceeds their physiologically acceptable level. In the case of mammalian cell isolation, cells are at risk of damage or death if they are suspended in a medium with a temperature above 310.15 K for an extended time [30,31]. The mutual restriction between various parameters in the scale-up process and the influence of uncontrollable Joule heating effect has become a major challenge in the development of DEP-based high-throughput microparticle separation platforms [32].

In our previous work, we studied the impact of Joule heating in an enlarged cIDE-DEP channel, in which an analytical solution based on a series of theoretical derivations was proposed with the buoyancy flow being considered as the dominating effect on particles’ DEP motion. However, the proposed model was only applicable for discontinuous DEP systems with cursory predictions [22]. Later, a continuous flow cIDE-DEP separator was constructed and a combined model based on a modified Lagrangian particle tracking solver that calculates the force fields according to Laplace’s equation and Navier–Stokes equation was validated experimentally by measuring motion trajectories of different sized polystyrene (PS) microparticles. Any thermal effect on both particles and fluid medium, however, was neglected in the developed model. To account for this simplification, an ideal suspension medium with very low conductivity (Milli-Q water) was adopted and a Peltier cooling plate was added into the separator to dissipate the generated heat. However, since the design of the separator based on an incomplete theoretical model was neither delicate nor precise, significant electric field-induced flow was still observed during the microparticle trajectory experiment [23]. The resultant thermal flow causes the PS particles to deviate from their original DEP-induced trajectory via hydrodynamic drag. Such uncontrollable particle trajectories caused by the thermal effect could not be accurately predicted through the developed model. This drawback, together with the induced device design deficiencies, such as excessive electrode numbers (26 electrodes), unoptimized outlet channels, as well as not fully functioning cooling pads, resulted in an unachievable separation of particles of interest.

In this work, we partially refer to the design concept of the separation channel adopted in our preliminary work and propose a novel hybrid floating cIDE separator. The new separator is composed of an optimized array of cIDE (7 electrodes in total) which act as working electrodes, in between which hybrid floating electrodes mediate the interference of Joule heating-induced fluid flow on DEP particle manipulation. A theoretical model to better predict microparticle trajectories in the proposed separator was established that incorporates the impact of both electrothermal and buoyancy effects on particles’ DEP motion. To evaluate the feasibility of the developed model and the performance of the separator, numerical simulations were carried out for the fractionation of different sized PS microparticles as the typical non-biological sample, and the separation of MDA-MB-231(Human breast cancer cells) from white blood cells (WBCs) and red blood cells (RBCs) as the representative bio-sample. Operating parameters that affect the separation performance were systematically studied with optimal isolation conditions for both PS microspheres and cells being screened out, respectively. To the best of the authors’ knowledge, research on the interference of Joule heating in continuous scaling-up DEP systems considering the effects of both electrothermal and buoyancy flows on DEP particle manipulation has not yet been reported. Additionally, the rational design of cIDE array with a well-defined ‘hybrid floating electrodes’ is, as far as we know, for the first time raised up in this work for attenuating the effect of temperature rise induced by Joule heating on bioparticles.

## 2. Design and Numerical Simulation

### 2.1. Working Principle

Generally, DEP-based separation (or fractionation) can be achieved due to differences in sample size and/or dielectric properties. Since the DEP force acting on particles is proportional to their volume, particles with different sizes can experience different magnitude of DEP forces. When particles are placed in the vicinity of an array of electrodes embedded at the bottom of a DEP channel, this causes differences in levitation height in case of negative DEP. This allows separation based on height differences of particles. Likewise, since the motion direction of particles depends on the difference in polarizability between the particle and the suspending medium, particles with different dielectric properties undergo different magnitude and/or even opposite directions of DEP force, giving rise to differences in levitation height, and therefore, possibilities of particle separation. The binary separation/isolation (for CTCs and blood cells) and ternary fractionation (for PS microspheres) in this study can thus be explained as particles of different dielectric properties and/or size are levitated to different heights due to the combined effects of DEP as well as Joule heating and hence being collected at different outlet channels.

### 2.2. Layout of the DEP Separator

**Figure 1:**
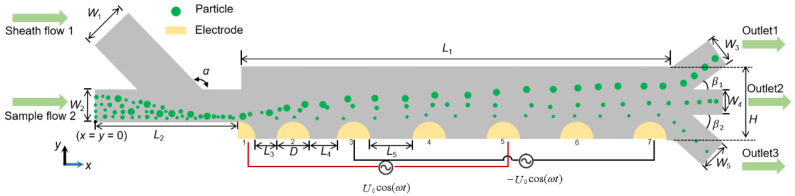
The layout of the hybrid floating cIDE separator (not drawn to scale).

The DEP separator is composed of two inlet channels (left side), three outlet channels (right side), and a particle separation chamber, as shown in Figure 1. The depth of the separator was specified as 2 cm. Detailed dimensions of the separator are shown in Table 1. Note that all given structure parameters have been numerically analyzed to ensure optimal separation performance.

**Figure A1:**
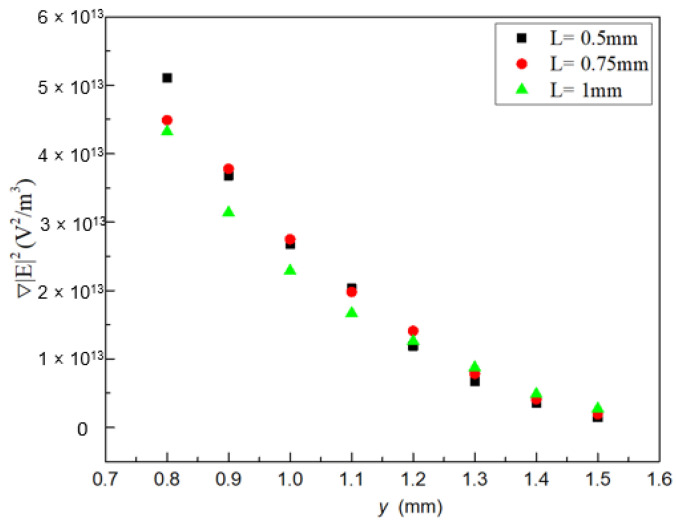
Dependence of the ∇|**E**|^2^ on height for three *D* = 1 mm electrode arrays with different spacing *L*.

The separation process of the particles can be roughly divided into three stages: the first stage (the inlet channel) is the indiscriminate focusing process of the particles. In this stage, the higher flow rate of the sheath flow auxiliary liquid focuses the particles to a single flow near the channel bottom. This focusing forces all particles to approach the electrode and have approximately identical initial position when entering the particle separation chamber, thereby facilitating the high-resolution particle separation. The second stage (the separation chamber) is the particle separation domain. In this part, an array of cIDE was used as the working electrode with hybrid floating electrodes in between to reduce the Joule heating interference. Floating electrodes are non-excited metal electrodes which are arrayed between two electrically excited electrodes being used to extend the DEP force [33]. The concept has demonstrated advantages in microsystems for the manipulation of particles, e.g., the use of floating electrode to maintain good DEP performance on membrane fouling mitigation with less energy input and hence alleviate the Joule heating problems [33]. The design of incremental electrode spacing was referred to our previous study which gives a sufficiently large electric field gradient in the vicinity of the electrode in the upstream domain while maintaining the effective working scope of the DEP force on particles in the downstream region [23], so that particles of different sizes/dielectric properties have sufficient difference in their levitation height (Figure A1). The third stage (the outlet channel) is the particle collection region, in which three outlets are arranged at specific heights, respectively, for collecting particles of interest, thereby achieving effective separation among particles.

### 2.3. Theory and Numerical Simulation

#### 2.3.1. Forces on Particles

When a suspended electrically neutral particle in the dielectric medium is in an external electric field, the charge within the particle will be rearranged under the action of the external electric field to form an induced dipole. If the particle is placed in a non-uniform electric field, the particle moves due to the imbalance of the forces acting on both sides of the particle, which is referred to as the DEP motion [34]. The movement direction of the particle mainly depends on the difference of polarizability between the particle and the suspending medium. When the polarizability of the particle is greater than that of the suspending medium, the particle is subjected to a positive DEP (pDEP) force, and its movement is towards maxima in the electric field. Conversely, when the polarizability of the particle is less than that of the surrounding medium, the particle will be moved away from field maxima by a negative DEP (nDEP) force. In the case of linear, isotropic dielectrics and when the dipole length is small, the time-averaged DEP force on a spherical particle can be expressed as [35]:

(1) FDEP=2πa3εmRe[K(ω)]∇|E|2

where *a* represents the radius of the spherical particle; $\varepsilon_{m}\;$ is the permittivity of the suspended medium; **E** is the electric field amplitude and Re[K(ω)] is the real part of Clausius–Mossotti factor. Re[K(ω)] gives the relative polarizability of the particle in the surrounding medium and dictates the movement of the particle in the non-homogeneous electric field. For homogeneous particles such as PS microspheres, K(ω) can be expressed by the composite permittivity of particles ($\varepsilon_{p}^{*}$) and suspending medium ($\varepsilon_{m}^{*}$):

(2) $$K\left(\omega \right)=\frac{\varepsilon_{p}^{*}-\varepsilon_{m}^{*}}{\varepsilon_{p}^{*}+2\varepsilon_{m}^{*}}\;\&\;\varepsilon^{*}=\varepsilon -j\sigma /\omega$$

Here, σ  is the conductivity and ω is the angular frequency of the applied electric field. For heterogeneous particles such as biological cells, a single-shell model is applied to describe the polarization properties more accurately. In this model, the core and the surrounding shell of a cell have different dielectric properties, which can be calculated by [36]:

(3) $$K\left(\omega \right)=\frac{\varepsilon_{c}^{*}-\varepsilon_{m}^{*}}{\varepsilon_{c}^{*}+2\varepsilon_{m}^{*}}$$

where the complex permittivity of the cell $\varepsilon_{c}^{*}$ can be expressed as:

(4) $$\varepsilon_{c}^{*}=\varepsilon_{2}^{*}\frac{{\left(\frac{a_{2}}{a_{1}}\right)}^{3}+2\left(\frac{\varepsilon_{1}^{*}-\varepsilon_{2}^{*}}{\varepsilon_{1}^{*}+2\varepsilon_{2}^{*}}\right)}{{\left(\frac{a_{2}}{a_{1}}\right)}^{3}-\left(\frac{\varepsilon_{1}^{*}-\varepsilon_{2}^{*}}{\varepsilon_{1}^{*}+2\varepsilon_{2}^{*}}\right)}$$

where $a_{1}$, $a_{2}$ are the radius of the interior inside the membrane and of the cell with membrane, respectively. $\varepsilon_{1}^{*}$ represents the complex permittivity of the interior, $\varepsilon_{2}^{*}$ is the complex permittivity of the cell membrane.

For frequencies below 1 MHz, Equation (4) simplifies to [12]:

(5)  Re[K(ω)]=f2−f02f2+2f02

Here, f is the frequency of the applied electric field, $f_{0}$ is the crossover frequency of the particles, which can be approximately expressed as: $f_{0}=\sigma_{m}/\pi aC_{\mathrm{mem}}$ with *C*_mem_, the membrane capacitance of the cell.

Due to the density difference between the particle and the suspension, particles are also subject to the gravitational force:

(6) $$F_{g}=\frac{4}{3}\pi a^{3}\left(\rho_{m}-\rho_{p}\right)g$$

Here, $\rho_{m}$ represents the density of the medium, $\rho_{p}$ represents the density of particles, and **g** represents the gravitational acceleration. In addition, the particles in the suspension are subject to fluid resistance, which can be expressed as:

(7) $$F_{\mathrm{drag}}=\frac{1}{\tau_{p}}m_{p}\left(u-v\right)\;\&\;\tau_{p}=\frac{2\rho_{p}a^{2}}{9\mu }$$

where $m_{p}$ represents mass of the particle, u and v are the velocity of fluid and particles, respectively, and μ is the dynamic viscosity of the fluid medium.

#### 2.3.2. Forces on the Fluid

In the process of dielectrophoretic particle separation, the Joule heating effect inevitably appears, which may cause changes in conductivity and permittivity of the suspension medium in DEP microsystems. Thus, the fluid is subject to a time-averaged electrothermal volume force (including Coulomb force and dielectric force), giving rise to micro vortex motion [37]:

(8) $$F_{e}=\frac{1}{2}\frac{\varepsilon_{m}\left(\alpha -\beta \right)}{1+\left(\omega \varepsilon_{m}/\sigma_{m}\right)}\left(\nabla T\cdot E\right)E-\frac{1}{4}\varepsilon_{m}\alpha_{m}{\left|E\right|}^{2}\nabla T$$

In this equation, α and β are the two thermal diffusion coefficients, which, in case of aqueous suspensions, can be expressed as: $\alpha =(\partial \varepsilon_{m}/\partial T)/\varepsilon_{m}\approx -0.04\;$(K^−1^),$\;\beta =(\partial \sigma_{m}/\partial T)/\sigma_{m}\approx$ 0.02 (K^−1^) [38]. Besides, in a separation system with a large volume or a high temperature rise, uneven Joule heating causes a temperature gradient inside the fluid, which induces local density differences and thus causes buoyancy flow. The buoyant volume force caused by Joule heating can be expressed as:

(9) $$F_{b}=\frac{\partial \rho_{m}}{\partial T}∆Tg$$

#### 2.3.3. Governing Equations and Boundary Conditions

The finite element analysis software COMSOL Multiphysics 5.5 is used to couple the physical fields of creeping flow, electric current, and fluid heat transfer. Flow field calculations are based on the continuity and the Navier–Stokes equations:

(10) $$\rho_{m}u\cdot \nabla u=-\nabla P+\nabla \cdot \left(\eta \nabla u\right)+F_{e}+F_{b}$$

(11) ∇u=0

Here, u indicates the fluid velocity field, *P* is the pressure, η is the dynamic viscosity of the fluid. The calculation of the electric field is based on the Laplace equation,

(12) $$\nabla^{2}\varphi =0$$

where E=−∇φ and  φ  represents the electric potential. The distribution of the electric field is induced by the potential exerted on the electrodes, *U*_0_, where there is $\varphi =\pm U_{0}$. The energy balance equation is adopted to link the electric field with the thermal field to solve the temperature distribution,

(13) $$\;k\nabla^{2}T+\frac{1}{2}\sigma_{m}E^{2}=0$$

where *k* denotes the thermal conductivity of the fluid medium [39]. The properties of selected materials are presented in Table A1.

Detailed boundary conditions (BC) used for simulations are given in Table A2. The computation domain in this study was considered to be two-dimensional since the electrodes are long compared to their diameter [40]. Note that an initial temperature of *T*_0_ = 293.15 K is given in the temperature field and an adiabatic BC is given to all channel walls and electrodes. This adiabatic BC instead of a constant heat flux is selected to simulate a worst-case study where we assume that there is no heat transfer from these boundaries. We believe that this assumption is practically reasonable since the materials for the proposed device fabrication, such as polyvinyl chloride (PVC) and polymethyl methacrylate (PMMA), are known as favorable thermal insulators.

#### 2.3.4. Model and Mesh-Independence Study

**Figure A2:**
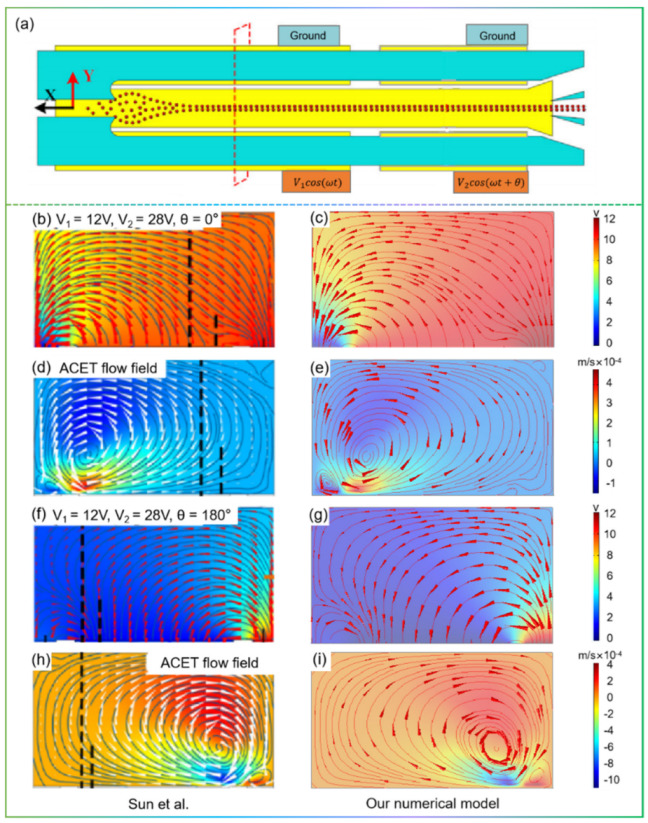
Model evaluation (validation of the electric field and the electrothermal flow): (**a**) Schematic diagram of the separator from Sun et al. (the simulated cross section is highlighted by the red dashed frame); Electric field comparison: (**b**,**f**) calculations from Sun et al. and (**c**,**g**) calculations using the proposed model from this work; Fluid flow comparison: (**d,h**) calculations from Sun et al. and (**e**,**i**) calculations using the proposed model from this work. Operating parameters: (**b**–**e**) u = 25 × 10^−6^ m/s, $\sigma_{m}\;$= 0.1 S/m, *f* = 1 MHz, V_1_ = 12 V, V_2_ = 28 V, θ = 0°; (**f**–**i**) u = 25 × 10^−6^ m/s, $\sigma_{m}$ = 0.1 S/m, *f* = 1 MHz, V_1_ = 12 V, V_2_ = 28 V, θ = 180°.

**Figure A3:**
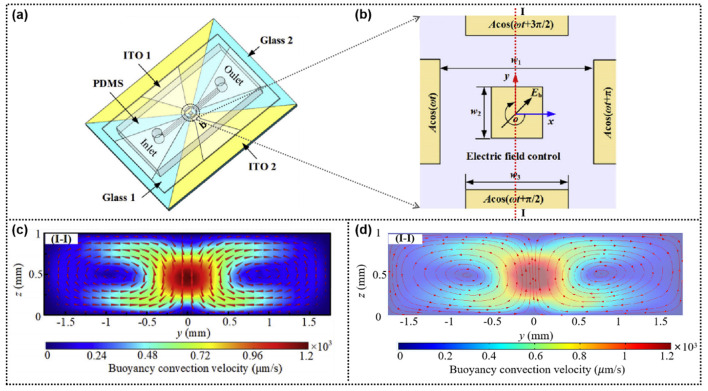
Model evaluation (validation of the buoyancy flow): (**a**) Schematic diagram of the separator from Zhang et al.; (**b**) electrodes arrangements (the simulated cross section is highlighted by the red dashed line I-I); Buoyancy flow at U_DC_ = 7 V: (**c**) calculations from Zhang et al. and (**d**) calculations using the proposed model from this work.

To verify that all model equations have been implemented correctly, the multi-physics coupling model used in this work was evaluated by numerical simulations of the electric and flow fields distributions from two recently published works. Based on the computational domain and BC given by Sun et al. [39] and Zhang et al. [29], the developed model was adopted for the electric field and the electrothermal flow (Figure A2), as well as the buoyancy flow simulations (Figure A3), respectively. The resultant electric field and both flow patterns are in good agreement with those in the literature, indicating the accuracy of the model used in this study.

**Figure A4:**
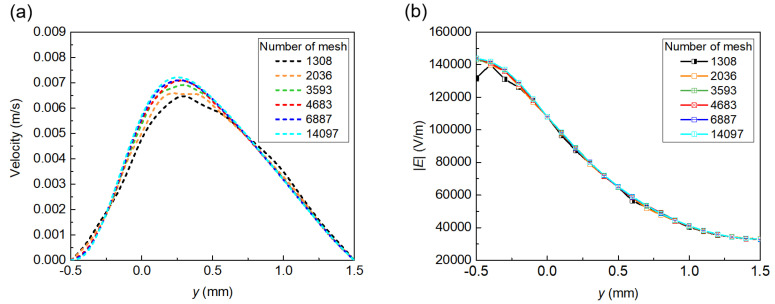
(**a**) Distribution of the flow velocity (**b**) Distribution of the electric field, for different numbers of elements: *x* = 15 mm, $u_{1}\;$= 2.5 mm/s, $u_{2}\;$= 1.5 mm/s, *U*_0_ = 152 V_eff_, $\sigma_{m}\;$= 1 S/m and *f* = 100 kHz.

A mesh-independence study was performed to obtain reliable simulation results that are independent of the mesh size (Figure A4). When the mesh number increased to 4683, the maximum relative error (the relative error in this case was calculated based on the ratio of the absolute error to the exact value, with the studied maximum mesh number 14,097 being considered as the exact value) of the fluid velocity and the electric field is 5% and 1%, with the mean relative error of 2% and 0.5%, respectively. Further increasing in the mesh number has little impact on the calculation results, nevertheless, it causes time-consuming computation. Therefore, the total mesh number in the computational domain was selected as 4683 with a minimum mesh size of 0.00615 mm in this study.

## 3. Results and Discussion

### 3.1. Distribution of Physical Fields in the Separator

**Figure 2:**
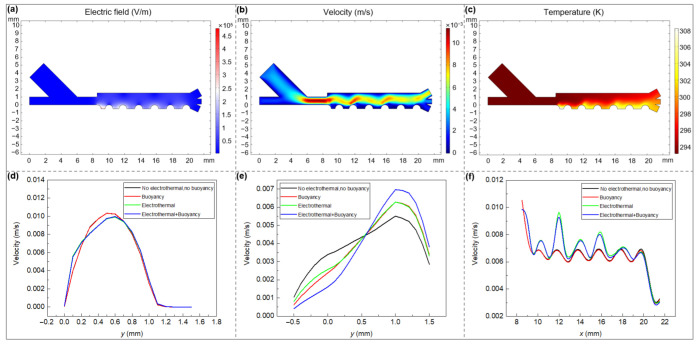
Numerical calculation of the distribution of: (**a**) Electric field, (**b**) Flow field, (**c**) Temperature field under an optimal operating condition that allows fractionation of PS particles: *U*_0_ = 152 V_eff_, *f* = 100 kHz, $\sigma_{m}$ = 0.001 S/m, $u_{1\;}$ = 2.5 mm/s, $u_{2}\;$= 1.5 mm/s; influence of electrothermal and buoyancy forces on the magnitude of the fluid velocity at different *x* and *y* positions: (**d**) *x* = 8.55 mm (**e**) *x* = 20.6 mm (**f**) *y* = 0.5 mm.

**Figure A5:**
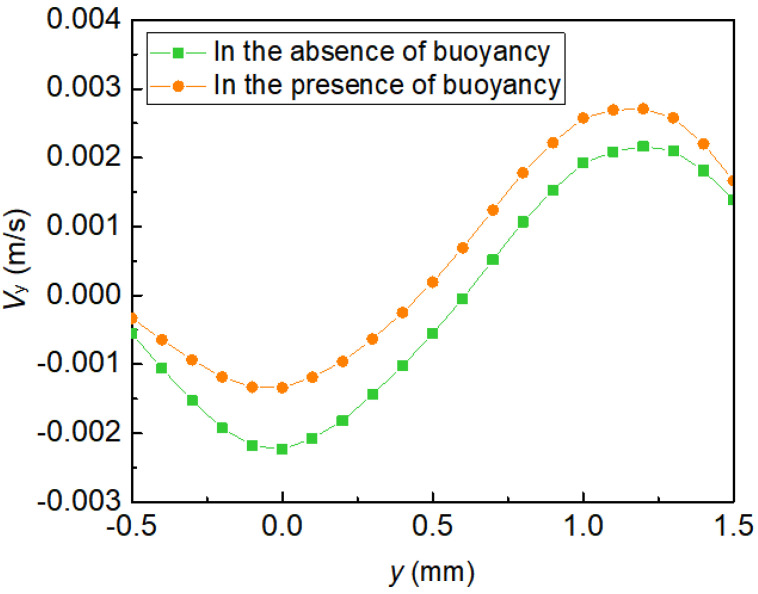
Velocity in the y-direction at the exit: *x* = 20.6 mm, *U*_0_ = 152 V_eff_, $u_{1}\;$= 2.5 mm/s, $u_{2}\;$= 1.5 mm/s, *f* = 100 kHz, $\sigma_{m}\;$= 0.001 S/m Velocity profile in the absence of buoyancy (green curve); Velocity profile in the presence of buoyancy (orange curve).

**Figure A6:**
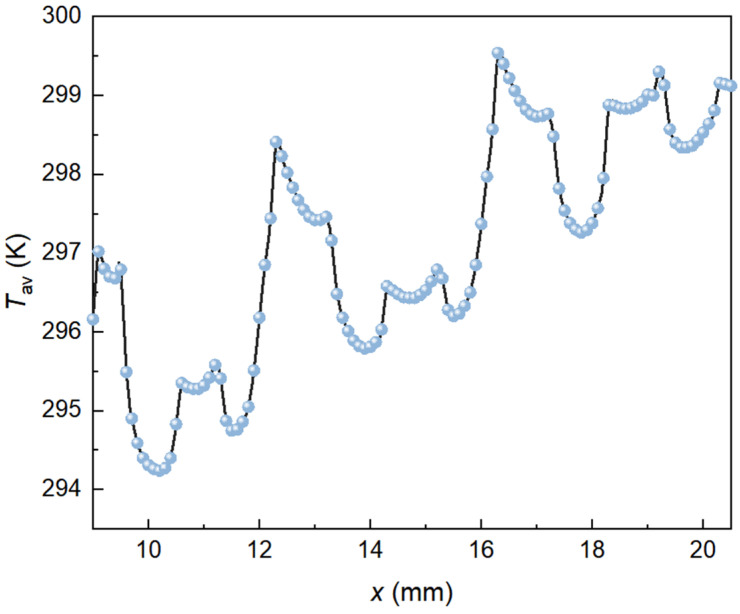
Variation of the temperature average on the *x* location.

The distributions of electric, flow and temperature fields within the separator are presented in Figure 2a–c. High electric fields are produced in the vicinity of electrodes with the maximum *E* appearing at the position in between the first pair of electrodes (Figure 2a). As expected, an electrode arrangement with different cIDE electrode spacings can ensure a maximum DEP force at the entry point of the separation channel for the most effective levitation of different particles in case of nDEP on the one hand, while maintaining a broad working range of DEP force for manipulating particles throughout the entire separation region on the other hand. For the fluid flow distribution shown in Figure 2b, the maximum flow velocity (11.36 mm/s) appears at the narrow region of the inlet channel where sample flow and sheath flow intersect. The fluid travels in waves instead of a smooth flow in the separation chamber primarily owing to the localized vortices around the electrodes that are caused by the electrothermal body force. The fluid presents obvious upward pointing flow near the outlet channel, which is due to the combined action of electrothermal and buoyancy effects. Different from electrothermal flow, the buoyancy flow demonstrates an anticlockwise global loop around the separation chamber in the continuous flow, where the position near the outlet is the upward flow part of the entire loop (Figure A5). The temperature profile shows the heat accumulation along the fluid flow direction (Figure A6). The highest temperature regions appear at positions around the electrodes and at the outlet channels with a maximum temperature of 307.9 K under simulated conditions (Figure 2c). Notably, the temperature value does not exceed the physiological temperature of cells, and the residence time of particles in the separation channel is very short in this study, i.e., around 10 s. Therefore, even if sensitive biological samples are processed through the separator, their physiological activity will not be greatly affected.

**Figure 3:**
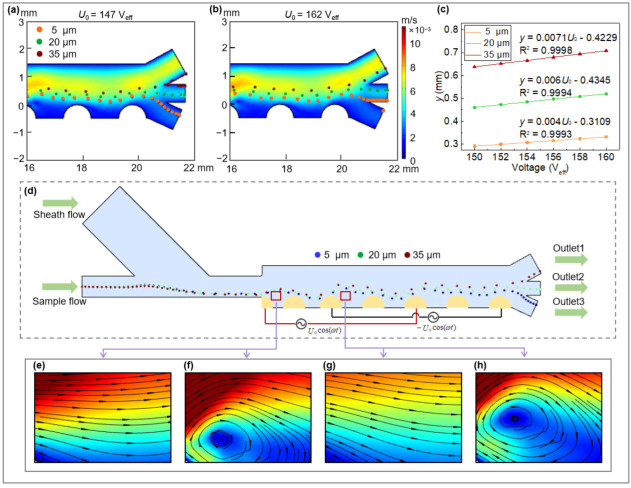
Effect of voltage on PS particle fractionation at $u_{1\;}$ = 2.5 mm/s, $u_{2}$ = 1.5 mm/s: (**a**,**b**) Fractionation of 5, 20 and 35-μm PS particles under the input voltage of (**a**) *U*_0_ = 147 V_eff_ and (**b**) *U*_0_ = 162 V_eff_ with velocity distribution as the background; (**c**) the dependence of applied voltage on levitation height of three different PS particles at *x* = 20.3 mm; (**d**) motion trajectories of three PS particles within the separator at *U*_0_ = 152 V_eff_; (**e**–**h**) fluid flow profile in the absence of the thermal field (**e**,**g**) and in the presence of the thermal field (**f**,**h**).

**Figure A7:**
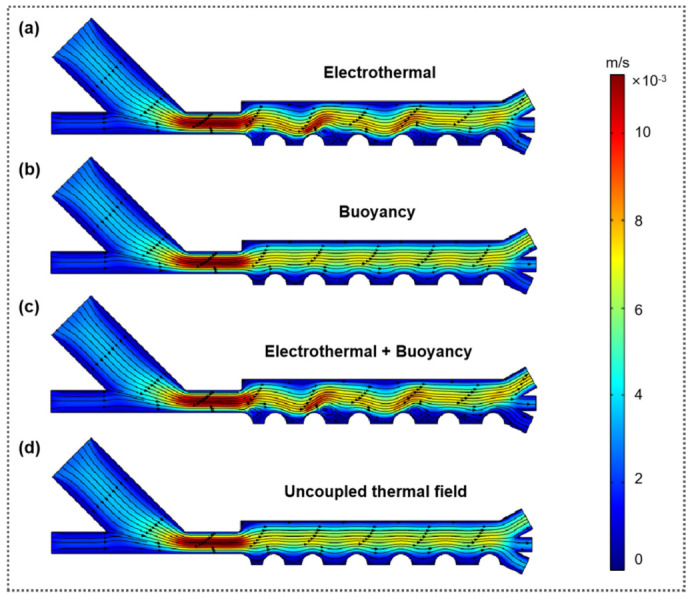
Comparison of the flow stream profile in continuous flow systems: (**a**) only electrothermal flow; (**b**) only buoyancy flow; (**c**) combined electrothermal and buoyancy flows; (**d**) uncoupled thermal field.

**Figure A8:**
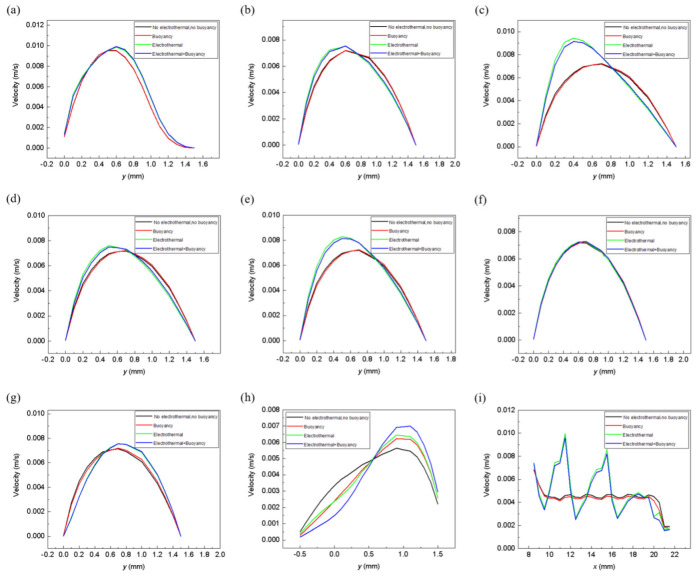
Influence of electrothermal and buoyancy forces on fluid flow in the separation channel: (**a**) *x* = 8.7 mm (electrode1: +*U*_0_) (**b**) *x* = 10.05 mm (electrode2: floating) (**c**) *x* = 11.8 mm (electrode3: −*U*_0_) (**d**) *x* = 13.8 mm (electrode4: floating) (**e**) *x* = 15.8 mm (electrode5: +*U*_0_) (**f**) *x* = 17.8 mm (electrode6: floating) (**g**) *x* = 19.8 mm (electrode7: −*U*_0_) (**h**) *x* = 20.5 mm (**i**) *y* = 0.2 mm.

We are specifically interested in the influence of the electrothermal and buoyancy forces on the fluid distribution in the continuous flow system (see Figure A7 for a comparison). In this work, the electrothermal and buoyancy effects on the fluid velocity under the study conditions are not apparent at the entrance of the separation channel (*x* = 8.55 mm) (Figure 2d). This is because the fluid disturbance which is based on the Joule heating-induced uneven temperature distribution inside the fluid is not obvious due to little heat accumulation at the beginning of the separation chamber. Except for regions near the electrode (below *y* = 0.7 mm), the electrothermal driven flows are dominating the fluid flow. However, the heat flow (including electrothermal flow and buoyancy flow) is negligible in the area close to the upper wall of the channel. In contrast, the heat accumulation near the exit (*x* = 20.6 mm) reaches the maximum value, resulting in the prominent heat flow disturbance over the entire height of the channel. Interestingly, four flow velocity lines intersect at the exit with the height of around *y* = 0.5 mm, at which both the electrothermal force and the buoyancy force have little effect on the fluid flow. Above this point, the velocities caused by electrothermal and buoyancy flow are predominant near the outlet channel, which exhibit nearly the same order of magnitude along the channel height direction (Figure 2e). To further explore this phenomenon, the heat flow velocity distribution along *x* direction at *y* = 0.5 mm is investigated (Figure 2f). It was found that in most cases, the overall flow velocity is more obviously influenced by electrothermal flow at this height compared to the buoyancy flow (the influences of electrothermal and buoyancy on flow velocity at other *y* positions within the separation channel are presented in Figure A8). That is, the localized vortex flow in the separation channel (see Figure 3f,h) is primarily based on temperature change of the fluid around the electrodes caused by the variations of electrical conductivity and dielectric constant. Moreover, four velocity lines almost overlap at the exit (*x* ≥ 20 mm), indicating a negligible contribution of electrothermal and buoyancy effects at the height of 0.5 mm, which is in accordance with the intersection point presented in Figure 2e. This may be attributed to an offset effect as electrothermal and buoyancy forces are similar in magnitude but opposite in direction. To sum up, when scaling-up DEP-based separation systems to enhance throughput, the influence of both electrothermal and buoyancy on the flow distribution, and hence, on the DEP particle manipulation should be considered to ensure accurate predictions.

### 3.2. Numerical Simulation of Non-Biological Particle Separation

In this section, PS microspheres are taken as the model particles to evaluate the separation effect of the proposed separator on a non-biological sample. The applicability of the separator in the continuous fractionation of differently sized PS was verified, and operating parameters that affect the fractionation were investigated.

#### 3.2.1. Fractionation of PS Microspheres

**Figure A9:**
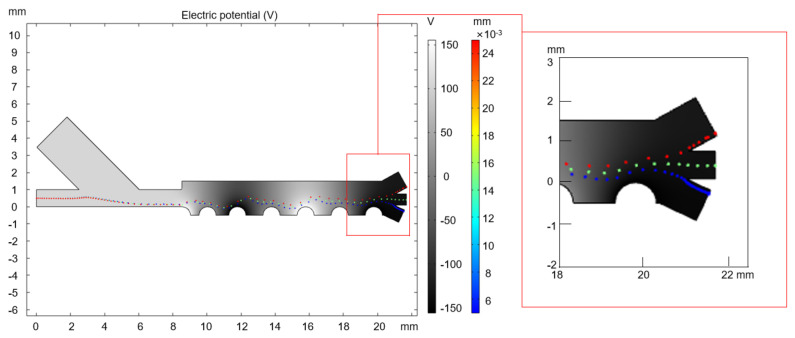
Separation of polystyrene samples based on dimensions (5 μm,15 μm, 25 μm): *U*_0_ = 156 V_eff_, *f* = 100 kHz, $\sigma_{m}\;$= 8 × 10^−4^ S/m, $u_{1}\;$= 2 mm/s $u_{2\;}$= 1 mm/s.

The feasibility of the separator was numerically demonstrated by the continuous fractionation of PS particles of three different sizes (5, 20 and 35 μm) under a sample throughput of 1.8 mL/min (based on the separator depth of 2 cm), as presented in Figure 3d. In the conductivity and frequency range investigated in this study, all PS microspheres were affected by nDEP forces and therefore moved away from the electrodes. Due to the cubic dependence of DEP force on particle radius, large particles experience stronger DEP force and thus move to higher positions, while smaller particles experience weaker DEP forces and hence are levitated to lower heights above the array. By adjusting the operating parameters, fractionation of PS with even smaller size differences (e.g., 5, 15 and 25 μm) is still possible (Figure A9).

#### 3.2.2. Impact of the Applied Voltage

**Figure A10:**
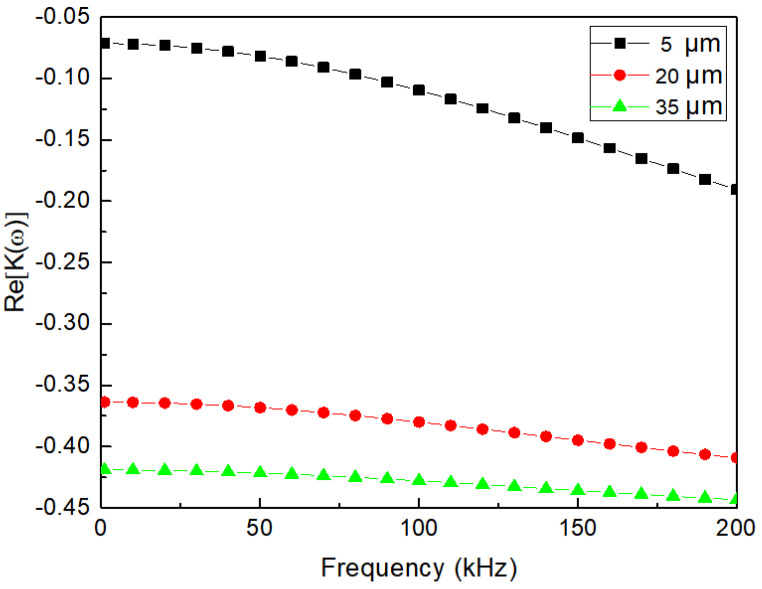
The Re[K(ω)] of PS over frequency under the medium conductivity of $\;\sigma_{m}=0.001\;S/m\;$.

**Figure A11:**
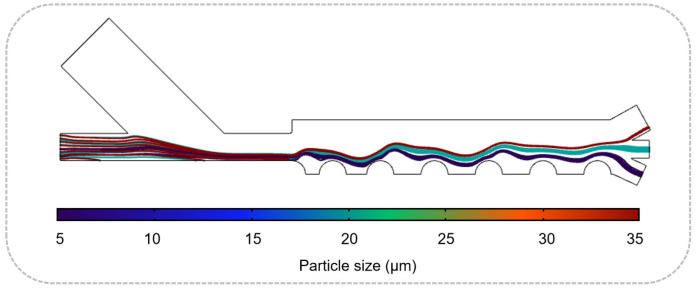
The dependence of different sized PS particle trajectories and final outlet positions on their initial injection positions: *U*_0_ = 152 V_eff_, *f* = 100 kHz, $\sigma_{m}\;$=0.001 S/m, $u_{1}\;$= 2.5 mm/s, $u_{2}\;$= 1.5 mm/s.

The dependence of PS fractionation on input voltage was studied using a medium conductivity of 0.001 S/m and an AC frequency of 100 kHz. In this case, all PS particles were subjected to negative DEP force (see Figure A10). When the input voltage (*U*_0_) was within the range of 148–160 V_eff_, full fractionation can be achieved (particle motion trajectories are illustrated in Figure 3d). Either low or high *U*_0_ outside this voltage range gives rise to undesired poor fractionation (Figure 3a,b). Within the effective voltage range, particle levitation height above the last electrode (*x* = 20.3 mm) increases linearly with the applied voltage (Figure 3c). The fits (Joule heating-induced thermal flow are included in this prediction) provide a guidance to facilitate more accurate control of particle position by varying the applied voltage, rendering the particle at a certain outlet or a specific suspension height in the separator. An increase in voltage not only consumes more energy, but also leads to an enhanced thermal effect ($W=\sigma_{m}E^{2}$). The uneven Joule heating causes electrothermal and/or buoyancy flow of the fluid and affects the DEP manipulation of the particle (Figure 3e–h). Note that the fluid around the electrodes has obvious vortex flow in the presence of the Joule heating (Figure 3f,h) as compared to those with smooth flow in the absence of the Joule heating (Figure 3e,g). Moreover, the dependence of PS particles’ trajectory line and final outlet position on their injection position were numerically evaluated. It was found that even if the particles are not injected from a single point, their trajectory lines in the separation channel are close to one another, and hence, they move through the same outlet channels (Figure A11).

#### 3.2.3. Impact of the Fluid Velocity at Inlet

**Figure 4:**
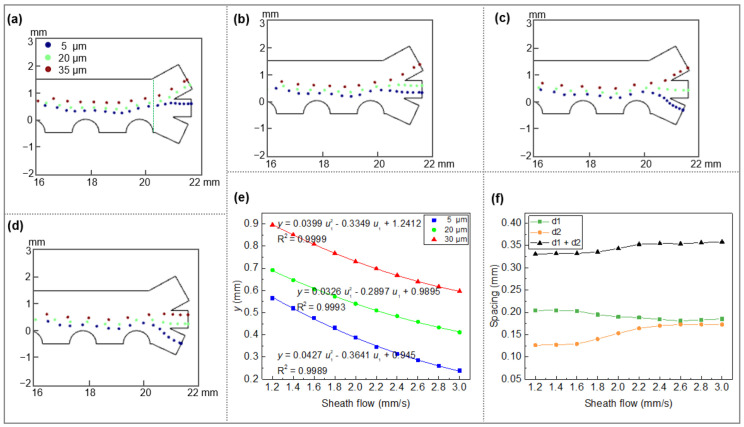
Fractionation of PS particles at different sheath flow (*U*_0_ = 152 V_eff_, *f* = 100 kHz, $\sigma_{m}$ = 0.001 S/m, $u_{2}$ = 1.5 mm/s): $u_{1}$ = (**a**) 1.5 mm/s, (**b**) 2 mm/s, (**c**) 2.5 mm/s, (**d**) 3 mm/s; (**e**) variation of particle height over sheath flow rate at *x* = 20.3 mm; (**f**) particle height spacing of different sizes at different velocity ratios (d1: the spacing between 20- and 35-μm PS; d2: the distance between 5- and 20-μm PS).

Flow velocities of both sheath flow $u_{1}$ and sample suspension flow $u_{2}$ were found to be key factors affecting the fractionation. The influences of the velocity ratio on the fractionation of PS microparticles were explored by varying $u_{1}$ from 1.5 to 3 mm/s while maintaining $u_{2}$ = 1.5 mm/s (Figure 4a–d). At $u_{1}$ = 2.5 mm/s, three different-sized PS particles can be collected at three outlet channels, respectively, demonstrating the successful fractionation of PS (Figure 4c). At low sheath flow velocity ($u_{1}$ = 1.5 mm/s), ternary fractionation cannot be achieved due to low levitation height differences of the three particles in the separation chamber, especially for 11-μm particles (Figure 4a). The height difference becomes broader at $u_{1}$ = 2 mm/s while the simultaneous fractionation of three particles can still not be achieved (Figure 4b). A similar situation appears at the sheath flow velocity of 3 mm/s (twice the suspension flow), in which the 35-μm particles flow toward the middle outlet channel because of the insufficient nDEP effect on those particles against the hydrodynamic force (Figure 4d).

To better guide the control of particle levitation height and thus the fractionation, the height of three differently sized PS particles near the outlet channel (*x* = 20.3 mm) were compared by varying the flow ratio (Figure 4e). For all particles, the levitation height decreases gradually with increasing the sheath flow velocity, where quadratic polynomial fittings were plotted to predict height position of three particles at the outlet. Moreover, the influence of sheath flow on height spacing among the three particles was investigated (Figure 4f). The height spacing between 35- and 20-μm PS particles (d1) reduces slightly (from 0.2 mm to 0.18 mm) with increasing the sheath flow velocity, whereas the height spacing between 20- and 5-μm PS particles (d2) enlarges. The overall height spacing (d1 + d2) varies slightly (in the range of 0.33–0.35 mm) at sheath flow velocities ranging from 1.8 to 2.2 mm/s, while it stays constant at other velocities. This small step-up of the overall height spacing at moderate sheath flow velocities may be attributed to the competitive effects between DEP and hydrodynamic drag, in which 11-μm particles are more likely to be affected by hydrodynamic drag as compared to 35-μm particles. Differently, particle motion is predominated by DEP and hydrodynamic drag at low and high sheath flow velocities, respectively.

### 3.3. Numerical Simulation of Bioparticle Separation

Different from previous sections where the fractionation of PS particles is based on size difference, the separation of biological cells demonstrated in this section depends mainly on difference in dielectric properties. When DEP is applied to biological particles instead of non-biological particles, additional factors need to be considered, not only to ensure effective manipulation, but also to pay attention to the bioactivity of the samples during the separation process.

#### 3.3.1. Frequency Response Characteristics of Cells

**Figure A12:**
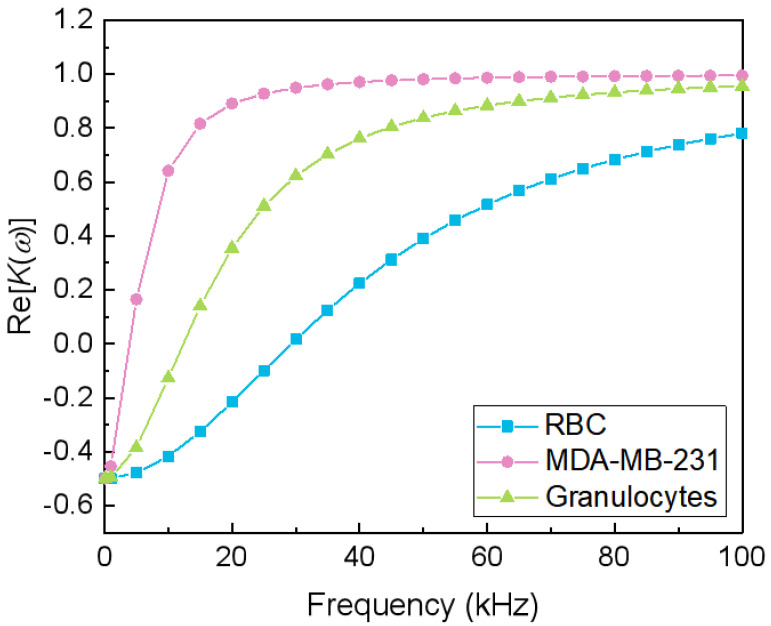
The dependence of  Re[K(ω)] of cells (RBC, WBC, CTC) on frequency at medium conductivity of $\sigma_{m}$= 0.002 S/m. All calculations are based on Equation (5) in the manuscript.

The applicability of the separator in biological particles separation was demonstrated by employing cells, i.e., RBC, WBC and MDA-MB-231 as representative particles, with cell properties being presented in Table 2. Considering the survival conditions of cells, the medium conductivity used in this study is 0.002 S/m. Previous studies have shown that cells can survive at this conductivity, and will not be damaged in structure or activity due to irreversible electroporation upon application of an electric field [32,41,42]. Figure A12 shows the Re[K(ω)] values of MDA-MB-231, RBC, and Granulocytes (a type of WBC) as a function of frequency for medium conductivities of 0.002 S/m. The separation of blood cells and MDA-MB-231 is possible over a wide frequency range under such medium conductivity. Note that the irreversible electroporation often occurs in electrokinetic system that may affect the bioactivity and damage the structure of cells in the form of an imposed transmembrane potential on cell membrane and causes membrane breakdown of cells, which becomes more prominent especially at systems with low medium conductivities. Using an approximation given by Voldman [43], the resulting imposed transmembrane potential under a medium conductivity of 0.002 S/m and an electric field strength of 7.75 × 10^4^ V/m (maximum electric field strength at which the majority of cells travel through) for MDA-MB-231 and RBCs is 167 and 257 mV, respectively, which is much lower than the threshold range of the transmembrane voltage (512–1028 mV, varying with different types of cells) estimated by Towhidi et al. [44] that leads to membrane electroporation and thus biochemical and physiological changes in the cell membrane. In this case, irreversible electroporation has very limited impact on cells, and hence, cell membrane electroporation will not happen.

#### 3.3.2. Influence of the Floating Electrodes

**Figure 5:**
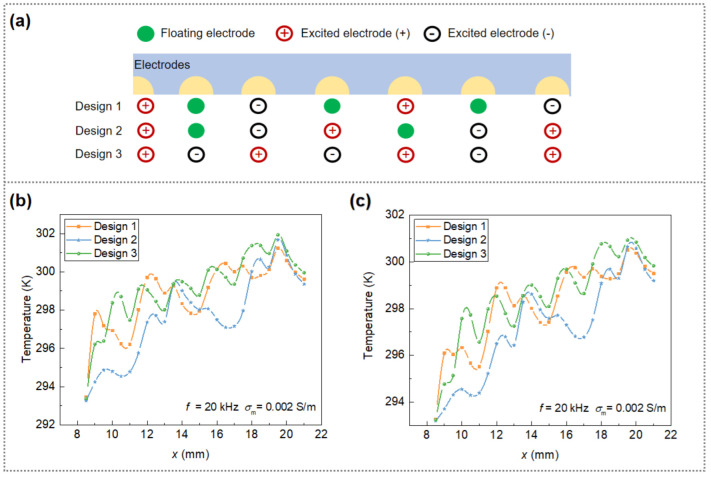
(**a**) Design strategies of the floating electrode arrangement; temperature profile at (**b**) *y* = 0.1 mm and (**c**) *y* = 0.2 mm for three different electrode designs.

Joule heating induced temperature rise in DEP-based systems is a very critical factor in case of biological sample separation, which cannot only affect the fluid flow and correspondingly the trajectory of particles, but also impact the biological activity or even damage the sample structure, especially when sensitive bioparticles are exposed to high temperatures for a long time. In this study, a floating electrode design was adopted in the cIDE separator to alleviate the interference of heat flow on cell separation as well as to reduce the energy consumption. The influence of the arrangement of floating electrodes on the cell separation was investigated with sample flow velocity and sheath flow of 1 and 1.5 mm/s, respectively. Three electrode arrays were studied with different designs: only floating interdigitated electrodes (one floating electrode between two electrically excited electrodes), design 1, hybrid interdigitated electrodes (floating electrode array connected with interdigitated electrode array), design 2, and only interdigitated electrodes (no floating electrode), design 3, as shown in Figure 5a. Voltages applied for the three different electrode array designs vary as *U*_0,1_ = 85 V_eff_, *U*_0,2_ = 59 V_eff_ and *U*_0,3_ = 46 V_eff_, respectively. This is to provide identical separation effects (i.e., for three electrode designs, the voltage is selected as a minimum value at which RBCs and MDA-MB-231 cells can be collected at different outlet channels) and is necessary due to the different distances between electrodes and hence different electric field strengths. Notably, although three different electrode designs give rise to different cell trajectories, the time for particles to travel through the whole separator (from inlet to outlet channel) is very close (i.e., around 10 s, in the case of $u_{1}$ = 1.5 mm/s and $u_{2}$ = 1 mm/s) irrelevant of electrode design. We focus our discussion on the temperature profiles at *y* = 0.1 mm and *y* = 0.2 mm since cells in most cases travel around these regions. As a result, the overall induced temperature rise in design 3 is the highest due to smallest spacing of the excitation electrodes which results in the strongest electric field, while design 2 presents the best temperature distribution along the channel. That is, design 2 exhibits the lowest temperature value in most cases despite a similar temperature profile of the three designs can be observed at the end of the channel (*x* = 19–22 mm). Moreover, design 2 has a maximum temperature rise of 8 K which effectively allows preventing the destruction of cell structure [30,31]. It should be noted that, on the one hand, the electrode design with floating electrodes (especially hybrid floating electrode as illustrated in Figure 5) can effectively reduce the temperature of the system, on the other hand, it requires a higher voltage input compared to designs without floating electrodes to achieve the same DEP separation effect. Generally, in DEP-based systems, the required input voltage increases with increasing number of floating electrodes so as to ensure sufficient DEP on particle manipulation. The problem is, however, excessive number of floating electrodes incorporated in a DEP system may inversely lead to more undesired energy dissipation and thus higher temperature rise within the system (e.g., see electrode design 1 and 2 for a comparison). To ensure the bioactivity of isolated cells while keeping the rise of temperature as low as possible, design 2 with the hybrid floating electrodes arrangement is used in cell separation studies.

#### 3.3.3. Effect of the Flow Velocity on Cell Separation

**Figure 6:**
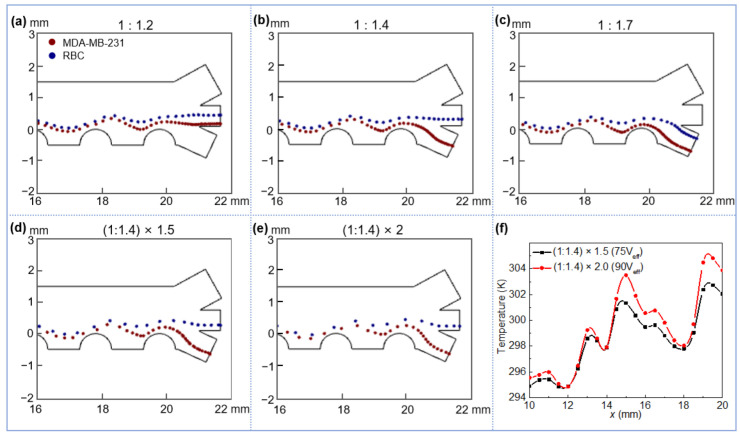
Separation of RBC and MDA-MB-231 at different flow ratios: (**a**) 1:1.2 (**b**) 1:1.4 (**c**) 1:1.7; the increase of both flow velocities by (**d**) 1.5 and (**e**) 2 times, respectively, while maintaining the same flow ratio of 1:1.4; (**f**) temperature profile at *y* = 0.1 mm (particle trajectory path) with increasing voltage and flow velocity.

In order to study the influence of the flow rate on cell separation, the voltage was fixed at 62 V_eff_ with an AC frequency of 20 kHz (separable voltage and frequency for RBCs and MDA-MB-231) and the sample flow was fixed at $u_{2}$ = 1 mm/s, the sheath flow ($u_{1})$ was adjusted to achieve different flow ratios (flow ratio $u_{2}:u_{1}$ ranging from 1:1.2 to 1:1.7). The isolation of MDA-MB-231 cells from RBCs is impossible under two extreme cases, i.e., minimum (1:1.2) and maximum (1:1.7) flow ratios, in which the two types of cells flow into the same outlet channel (Figure 6a,c). At very low sheath flow velocity, Joule heating-induced upward flow dominates the overall flow direction near the outlet, resulting in the deflection of MDA-MB-231 cells against pDEP effect toward the middle outlet channel (Figure 6a). On the contrary, the residence time of RBCs with respect to nDEP effect is insufficient at very high sheath flow velocity, giving rise to a limited levitation height of RBCs which are then directed to the lower outlet channel (Figure 6c). The isolation between two cells is possible at moderate sheath flow velocities (the separable $u_{1}$ was numerically confirmed to range from 1.3–1.6 mm/s with an optimal value of 1.4 mm/s) as illustrated in Figure 6b. Interestingly, by increasing the operating voltage to 75 and 90 V_eff_, cell separations are still feasible at 1.5 and 2 times higher flow velocities (Figure 6d,e) with allowable temperature rises for cell survival (see Figure 6f, the maximum temperature is around 305 K, which is lower than the threshold temperature of 311 K that may affect cell physiological activity [43]), indicating the possibility of further improving the throughput of the separator towards clinical applications.

**Figure 7:**
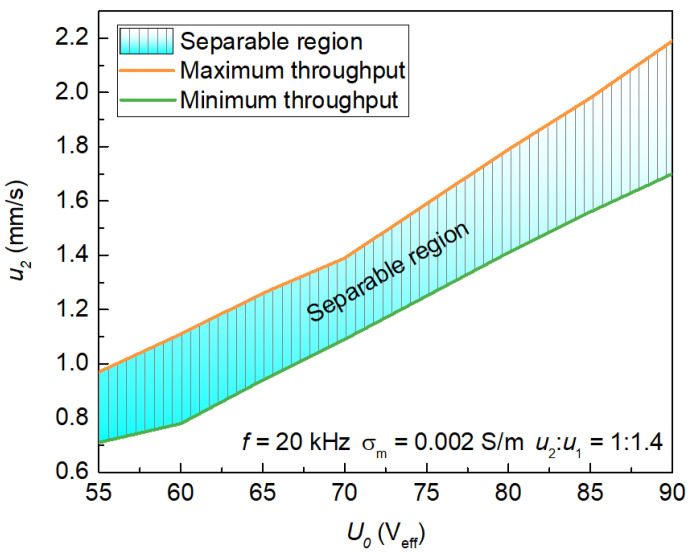
Calculated system processing capability of the hybrid floating cIDE separator at different driving voltage.

To explore the potential of throughput improvement for the proposed separator, the range of sample velocity ($u_{2}$) that can be processed at various excitation voltages ($U_{0}$) was analyzed. Figure 7 shows the separation of RBC and MDA-MB-231 cells under the driving voltages that allow temperature rise still within the cell physiological activity range, in which the striped area indicates the separable region of cells with top and bottom curves representing upper and lower limits of the device throughput, respectively. At regions above the maximum throughput, sample separation cannot be achieved due to insufficient residence time of cells for levitation height-based differentiation within the separator which is caused by the excessive fluid flow in horizontal direction. At regions below the minimum throughput, high voltage induced a strong DEP force as well as vortex flow at relatively low horizontal flow velocity gives rise to uncontrollable motion trajectories of cells either being trapped at the electrode (for MDA-MB-231 having pDEP effect) or moving to the same outlet channels (for both cells), thereby separation is not possible under this circumstance. Under the allowable voltage range, the system processing capacity increases with increasing applied voltage, and the optimal throughput of the system can thus be estimated at a given voltage. On the one hand, the increment of voltage allows cell separation at higher flow rate, thereby improving the system’s throughput. On the other hand, with the increase of the flow rate, the DEP velocity must also increase to overcome the flow velocity to allow the effective separation of cells. Using this as a basis, appropriate voltage and flow rate can be selected in accordance with specific separation requirements. Notably, for the isolation of MDA-MB-231 cells from RBC in this case, the maximum sample throughput of the proposed separator is calculated to be 2.6 mL/min under an applied voltage of 90 V_eff_.

#### 3.3.4. Effect of Joule Heating-Induced Fluid Flow on Cell Separation

**Figure 8:**
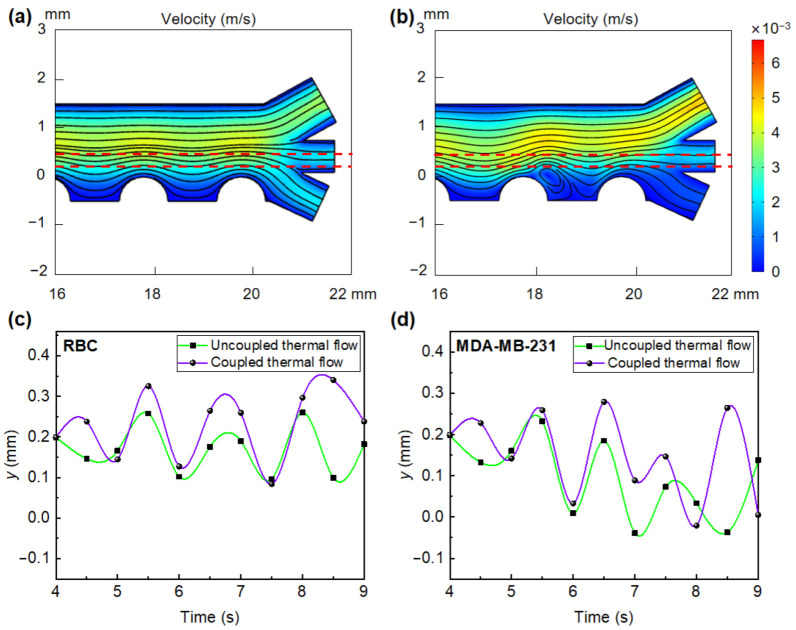
The influence of heat flow induced by Joule heating on cell separation process at *U*_0_ = 60 V_eff_ (electrode array design 2): (**a**) Fluid flow in an uncoupled thermal field; (**b**) fluid flow in a coupled thermal field; height difference of (**c**) RBCs and (**d**) MDA-MB-231 in the separation channel with coupled/uncoupled thermal effect.

**Figure A13:**
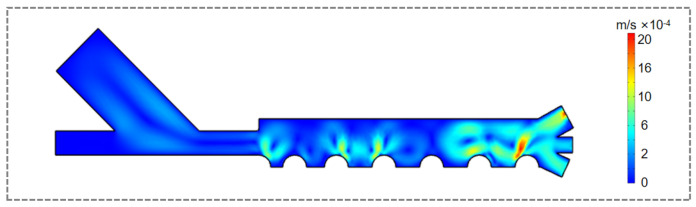
Distribution of heat flow in hybrid floating cIDE separator under zero initial velocity ($u_{1}$= 0, $u_{2}$= 0).

**Figure A14:**
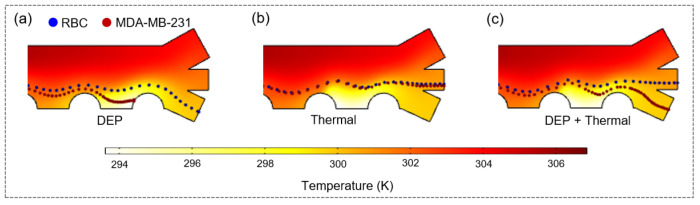
The trajectory of RBCs and MDA-MB-231 with temperature distribution as the background: *U*_0_ = 62 V_eff_, $u_{1}$= 1.4 mm/s, $u_{2}$= 1 mm/s, *f* = 20 kHz, $\sigma_{m}\;$= 0.002 S/m: (**a**) Only under the action of DEP force; (**b**) Only under the influence of heat flow; (**c**) Under the combined action of DEP force and heat flow.

The existence of Joule heating-induced heat flow was found to affect the original fluid flow within the separator. Local vortices can be observed in the vicinity of the electrodes when considering the effect of Joule heating in the simulation (Figure 8b), as compared to an evenly distributed fluid profile in the absence of Joule heating (Figure 8a). Specifically, in the particle trajectory region (marked with red dashed line), as shown in Figure 8a,b, streamlines that go into the lower channel in case of uncoupled thermal field will be directed towards the middle outlet in case of coupled field. In addition, the influences of Joule heating on the flow distribution at zero initial velocity of the fluid were explored, in which vortex flows around the electrodes have been found. The fluid flow caused by Joule heating near the outlet is particularly obvious, which is based on a combined effect of electrothermal and buoyancy under high temperature differences, forming a distinct upward directed flow at the outlet (Figure A13). This upward heat flow from the bottom of the separator has a greater effect on small particles, thus, RBCs that have a smaller size (2.5 μm) than MDA-MB-231 (6.2 μm) are more likely to shift upward in the presence of the biased flow. The upward motion of RBCs caused by heat flow is in the same direction as the movement through nDEP force under the frequency of 20 kHz. MDA-MB-231 cells are mainly affected by pDEP force at this frequency, while the existence of heat flow promotes them to drift upward, to avoid the direct contact of sensitive bioparticles to the electrodes. The trajectories of RBC and MDA-MB-231 cells in the coupled/uncoupled heat flow are plotted over time, respectively (Figure 8c,d). It can be found that RBCs and MDA-MB-231 are levitated to higher positions by coupling the heat flow as compared to that without heat flow. In this case, Joule heating-induced heat flow enhances the nDEP force. Moreover, the motion trajectories of RBCs and MDA-MB-231 in the separator further confirm the combined effect of the heat flow and the DEP force on cell separation (Figure A14).

#### 3.3.5. Isolation of MDA-MB-231 from Granulocytes and Separation of Blood Cells

**Figure 9:**
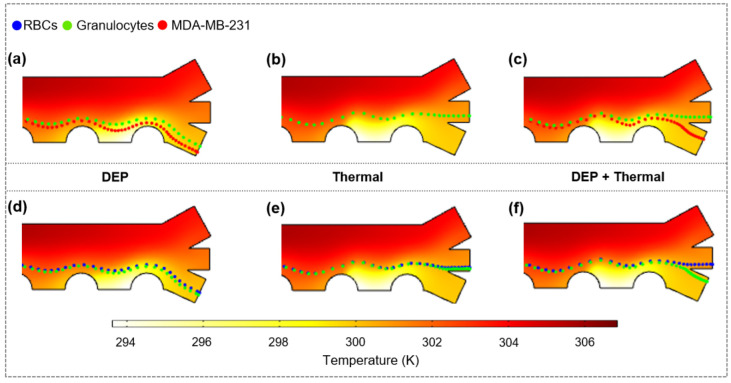
Simulated isolation of MDA-MB-231 from Granulocytes (*U*_0_ = 62 V_eff_, *f* = 10 kHz) as well as separation of RBCs and Granulocytes (*U*_0_ = 59 V_eff_, *f* = 25 kHz) with temperature distribution as backgrounds. (**a**,**d**) Only DEP force; (**b**,**e**) only heat flow; (**c**,**f**) combined effects of DEP and heat flow. $u_{1}$ = 1.4 mm/s, $u_{2}$ = 1 mm/s, $\sigma_{m}$ = 0.002 S/m.

The isolation of MDA-MB-231 from Granulocytes as well as the separation of RBCs and Granulocytes was numerically demonstrated through the proposed separator. The AC frequencies for both separation tasks were selected based on reverse DEP effects of cells to be separated (see Figure A12). Neither DEP force (Figure 9a,d) nor thermal flow (Figure 9b,e) can achieve separation/isolation when they act on cells separately, while they are successfully separated under the combined action of DEP as well as electrothermal and buoyancy-induced hydrodynamic drag on cells (Figure 9c,f). Due to a complete overlap of both cell motion trajectories, only Granulocytes can be observed in Figure 9b, implying that the heat flow has negligible impact on trajectory differences of Granulocytes and MDA-MB-231. Notably, the effect of DEP did not make the significant difference between the trajectories of RBCs and Granulocytes; Only a noticeable difference was found in the vicinity of the outlet channels, indicating that RBCs and Granulocytes separation depends more on the heat flow.

## 4. Conclusions

In summary, we reported a novel design of the hybrid floating cIDE separator for continuous high-throughput separation of biological and abiotic microparticles. The separator was composed of two inlet channels and three outlet channels as well as a separation chamber that allow sheath flow-assisted ternary particle separation, in which the separation chamber was assembled with an array of cIDE incorporating hybrid floating electrode layout. A theoretical model by coupling the electric field, flow field and temperature field was established to predict microparticle trajectories that are influenced by Joule heating induced electrothermal and buoyancy effects in a scale-up DEP system. Numerical simulations were performed to demonstrate the accuracy of the theoretical model and the feasibility of the proposed separator. For the proof-of-concept study, standard PS microspheres were employed as typical non-bioparticles, and RBCs, Granulocytes, and MDA-MB-231 cells were adopted as representative bioparticles. Size-based separation was numerically demonstrated through the floating cIDE separator for the effective fractionation of 5, 20, and 35-μm PS microparticles with a sample throughput up to 1.8 mL/min. Depending on different electric properties of cells, the separation of RBCs and Granulocytes and the isolation of CTCs from RBCs were simulated, respectively, by using the separator, realizing the successful collection of cells of interest with desirable sample throughputs as high as 2.6 mL/min. The heat flow caused by localized temperature difference due to Joule heating was confirmed to affect particle’s DEP motion in the continuous flow system, especially at positions close to the electrodes and the outlet channels. Interestingly, such heat flow which is capable of deflecting particles in upward direction, in some cases, promotes the DEP separation. Moreover, the design of floating electrodes arrangement as well as system operating parameters were numerically investigated to allow better separation under Joule heating interference. Moreover, it was found that the temperature rise in such DEP system has negligible impacts on bioactivity of cells, which further confirms the capability of this versatile separator for the continuous separation of sensitive bioparticles. Overall, this work is expected to build a bridge between the separation principles followed by the micrometer-level chips and the millimeter-level separators, providing a design reference to further improve the throughput of DEP-based platforms. Future work may consider device fabrication and experimental verification of the separation performance of the hybrid floating cIDE separator.

## Figures and Tables

**Table 1:** Specification of characteristic dimensions of the separator.

Parameters	*L*_1_	*L*_2_	*L*_3_	*L*_4_	*L*_5_	*W*_1_	*W*_2_	*W*_3_	*W*_4_	*W*_5_	*H*	*D*	*α*	*β*_1_	*β*_2_
Value (mm)	12.5	8.53	0.5	0.75	1	2.5	1	1.05	0.66	0.8	2	1	135°	30°	25°

**Table 2:** The types and properties of selected cells.

Cell Type	Radius (μm)	Membrane Capacitance (mF/m^2^)	References
Red blood cells	2.5	8.7	[41]
Granulocytes	4.71 ± 0.23	11.0 ± 3.2	[12]
MDA-MB-231	6.2 ± 0.58	25.9 ± 4.2	[12]

## Data Availability

Not applicable.

## Appendix A

**Table A1:** Summary of the material properties used in the model.

	PS	RBCs	Granulocytes	MDA-MB-231	Fluid
Diameter (µm)	5, 20, 35	5	9.42 ± 0.46	12.4 ± 1.16	
Density (kg/m^3^)	1050	1050	1050	1050	1000
Dynamic viscosity (Pa·S)					1 × 10^−3^
Conductivity (S/m)	8 × 10^−4^, 2 × 10^−4^, 1.143 × 10^−4^	0.31	0.6	0.62	0.001, 0.002
Permitivity	2.55	59	151	52	80
$\sigma_{\mathrm{mem}}$ (S/m)		1 × 10^−6^	1 × 10^−6^	1 × 10^−6^	
$\mu_{\mathrm{men}}$		4.44	5	11.75	
Membrane thickness (nm)		9	4	4	

**Table A2:** Computation domain and boundary conditions employed in COMSOL Multiphysics.

Electric Currents Module	Element	Definition
Current conservation	Whole domain	$\nabla \cdot J=Q_{J,V}\;J\;=\;\sigma E+J_{e}\;E\;=-\nabla V$
Initial values	Whole domain	
Electric insulation	Wall	n·J=0
Electric potential 1	Electrodes 1, 5	$U_{0}$
Electric potential 2	Electrodes 3, 7	$-U_{0}$
Suspended potential	Electrodes 2, 4, 6	$\int_{\partial \Omega }^{\;}-n\cdot Jds=I_{0}$
Laminar flow module		
Inlet	Inlet 1, 2	$u_{1},u_{2}$
Outlet	Outlet 1, 2, 3	P=0
No slip	Wall, Electrodes	u=0
Gravity	Whole domain	
Body force	Whole domain	$F_{e}=\frac{1}{2}\cdot \frac{\varepsilon_{m}\left(\alpha -\beta \right)}{1+\left(w\varepsilon_{m}/\sigma_{m}\right)}\left(\nabla T\cdot E\right)E-\frac{1}{4}\varepsilon_{m}\alpha_{m}{\left|E\right|}^{2}\nabla T$
Fluid heat transfer module		
Inlet	Inlet 1, 2	$-n\cdot q=d_{z}\rho ∆Hu\cdot n$
Outlet	Outlet 1, 2, 3	−n·q=0
Thermal insulation	Wall, Electrodes	−n·q=0
Particle tracking module		
Inlet	Inlet 2	$q=q_{0}$ $v=v_{0}$
Outlet	Outlet1, 2, 3	
Drag force	Whole domain	$F_{D}=\frac{1}{\tau_{P}}m_{P}\left(u-v\right)\;\tau_{P}=\frac{\rho_{P}d_{P}^{2}}{18\mu }$
Dielectrophoresis	Whole domain	$F_{DEP}=2\pi a^{3}\varepsilon_{m}Re\left[k\left(\omega \right)\right]\nabla {\left|E\right|}^{2}$

The conductivity of PS particle can be calculated according to the following equation: $\sigma_{p}\;$= 2 *K*_s_/a, where *K*_s_ = 1 × 10^−9^ S represents the surface conductivity, a is the radius of the particle, thus the conductivity of PS particles with a radius of 2.5 μm, 10 μm and 17.5 μm used in this paper is $\sigma_{p1}\;$= 8 × 10^−4^ S/m, $\sigma_{p2}\;$= 2 × 10^−4^ S/m, $\sigma_{p3}\;$= 1.143 × 10^−4^ S/m respectively.

The real part of the Clausius-Mosotti factor Re[K(ω)] of PS at different frequencies can be approximated by: Re[K(ω)]=(σp−σm)(σp+2σm)+(2πf)2ε02(εp−εm)(εp+2εm)(σp+2σm)2+(2πf)2ε02(εp+2εm)2, the direction of dielectrophoresis force on particles can be obtained to guide better separation.

## References

[1] Pesch G.R., Du F. (2020). A review of dielectrophoretic separation and classification of non-biological particles. Electrophoresis.

[2] Jia Y.K., Ren Y.K., Jiang H.Y. (2015). Continuous dielectrophoretic particle separation using a microfluidic device with 3D electrodes and vaulted obstacles. Electrophoresis.

[3] Ballantyne G.R., Holtham P.N. (2014). Evaluation of the potential for using dielectrophoresis to separate minerals. Miner. Eng..

[4] Kwak T.J., Jung H.H., Allen B.D., Demirel M.C., Chang W.J. (2021). Dielectrophoretic separation of randomly shaped protein particles. Sep. Purif. Technol..

[5] Ghomian T., Jeong H., Pan V., Celik K., Alangari M., Ke Y.G., Hihath J. (2021). High-Throughput Dielectrophoretic Trapping and Detection of DNA Origami. Adv. Mater. Interfaces.

[6] Han P., Yosinski S., Kobos Z.A., Chaudhury R., Lee J.S., Fahmy T.M., Reed M.A. (2020). Continuous Label-Free Electronic Discrimination of T Cells by Activation State. ACS Nano.

[7] Adams T.N.G., Jiang A.Y.L., Mendoza N.S., Ro C.C., Lee D.H., Lee A.P., Flanagan L.A. (2020). Label-free enrichment of fate-biased human neural stem and progenitor cells. Biosens. Bioelectron..

[8] Liu Y., Hayes M.A. (2020). Orders-of-Magnitude Larger Force Demonstrated for Dielectrophoresis of Proteins Enabling High-Resolution Separations Based on New Mechanisms. Anal. Chem..

[9] Kung Y.C., Niazi K.R., Chiou P.Y. (2020). Tunnel dielectrophoresis for ultra-high precision size-based cell separation. Lab Chip.

[10] Cao Z., Zhu Y., Liu Y., Dong S.R., Chen X., Bai F., Song S.X., Fu J.X. (2018). Dielectrophoresis-Based Protein Enrichment for a Highly Sensitive Immunoassay Using Ag/SiO_2_ Nanorod Arrays. Small.

[11] Barik A., Zhang Y., Grassi R., Nadappuram B.P., Edel J.B., Low T., Koester S.J., Oh S.H. (2017). Graphene-edge dielectrophoretic tweezers for trapping of biomolecules. Nat. Commun..

[12] Derakhshan R., Ramiar A., Ghasemi A. (2020). Numerical investigation into continuous separation of particles and cells in a two-component fluid flow using dielectrophoresis. J. Mol. Liq..

[13] Jones P.V., Salmon G.L., Ros A. (2017). Continuous Separation of DNA Molecules by Size Using Insulator-Based Dielectrophoresis. Anal. Chem..

[14] Zhang Y., Wang S.Y., Chen J., Yang F., Li G.Y. (2020). Separation of Macrophages Using a Dielectrophoresis-Based Microfluidic Device. Biochip J..

[15] Xie Y.L., Rufo J., Zhong R.Y., Rich J., Li P., Leong K.W., Huang T.J. (2020). Microfluidic Isolation and Enrichment of Nanoparticles. ACS Nano.

[16] Zhang X.Z., Xu X.W., Ren Y., Yan Y.Y., Wu A.G. (2021). Numerical simulation of circulating tumor cell separation in a dielectrophoresis based Y-Y shaped microfluidic device. Sep. Purif. Technol..

[17] Huang X., Torres-Castro K., Varhue W., Salahi A., Rasin A., Honrado C., Brown A., Guler J., Swami N.S. (2021). Self-aligned sequential lateral field non-uniformities over channel depth for high throughput dielectrophoretic cell deflection. Lab Chip.

[18] Nie X.F., Luo Y., Shen P.H., Han C.W., Yu D.L., Xing X.X. (2021). High-throughput dielectrophoretic cell sorting assisted by cell sliding on scalable electrode tracks made of conducting-PDMS. Sens. Actuators B Chem..

[19] Aghaamoo M., Aghilinejad A., Chen X.L., Xu J. (2019). On the design of deterministic dielectrophoresis for continuous separation of circulating tumor cells from peripheral blood cells. Electrophoresis.

[20] Li Y., Wang Y., Wan K., Wu M., Guo L., Liu X., Wei G. (2021). On the design, functions, and biomedical applications of high-throughput dielectrophoretic micro-/nanoplatforms: A review. Nanoscale.

[21] Wang Y., Du F., Baune M., Thoming J. (2014). Dielectrophoresis in aqueous suspension: Impact of electrode configuration. Microfluid. Nanofluid..

[22] Wang Y., Du F., Baune M., Thoming J. (2015). Predicting and eliminating Joule heating constraints in large dielectrophoretic IDE separators. Chem. Eng. Sci..

[23] Wang Y., Du F., Pesch G.R., Koser J., Baune M., Thoming J. (2016). Microparticle trajectories in a high-throughput channel for contact-free fractionation by dielectrophoresis. Chem. Eng. Sci..

[24] Calero V., Garcia-Sanchez P., Honrado C., Ramos A., Morgan H. (2019). AC electrokinetic biased deterministic lateral displacement for tunable particle separation. Lab Chip.

[25] Salari A., Navi M., Dalton C. (2015). A novel alternating current multiple array electrothermal micropump for lab-on-a-chip applications. Biomicrofluidics.

[26] Dalili A., Montazerian H., Sakthivel K., Tasnim N., Hoorfar M. (2021). Dielectrophoretic manipulation of particles on a microfluidics platform with planar tilted electrodes. Sens. Actuators B Chem..

[27] Du F., Baune M., Thoming J. (2007). Insulator-based dielectrophoresis in viscous media—Simulation of particle and droplet velocity. J. Electrostat..

[28] Ramos A., Morgan H., Green N.G., Castellanos A. (1998). Ac electrokinetics: A review of forces in microelectrode structures. J. Phys. D Appl. Phys..

[29] Zhang K., Ren Y., Tao Y., Deng X., Liu W., Jiang T., Jiang H. (2020). Efficient particle and droplet manipulation utilizing the combined thermal buoyancy convection and temperature-enhanced rotating induced-charge electroosmotic flow. Anal. Chim. Acta.

[30] Mittal N., Rosenthal A., Voldman J. (2007). NDEP microwells for single-cell patterning in physiological media. Lab Chip.

[31] Aghilinejad A., Aghaamoo M., Chen X.L., Xu J. (2018). Effects of electrothermal vortices on insulator-based dielectrophoresis for circulating tumor cell separation. Electrophoresis.

[32] Ji J.L., Wang J.X., Wang L., Zhang Q., Duan Q.Q., Sang S.B., Huang Q., Li S.S., Zhang W.D., Jiang X.N. (2020). Dynamic-coupling analyses of cells localization by the negative dielectrophoresis. Proc. Inst. Mech.Eng. Part C J. Mech. Eng. Sci..

[33] Du F., Hawari A.H., Larbi B., Ltaief A., Pesch G.R., Baune M., Thoming J. (2018). Fouling suppression in submerged membrane bioreactors by obstacle dielectrophoresis. J. Membr. Sci..

[34] Turcan I., Olariu M.A. (2020). Dielectrophoretic Manipulation of Cancer Cells and Their Electrical Characterization. ACS Comb. Sci..

[35] Kwizera E.A., Sun M., White A.M., Li J., He X. (2021). Methods of Generating Dielectrophoretic Force for Microfluidic Manipulation of Bioparticles. ACS Biomater. Sci. Eng..

[36] Abdulla A., Zhang T., Ahmad K.Z., Li S., Lou J., Ding X. (2020). Label-free Separation of Circulating Tumor Cells Using a Self-Amplified Inertial Focusing (SAIF) Microfluidic Chip. Anal. Chem..

[37] Sun H.Z., Ren Y.K., Hou L.K., Tao Y., Liu W.Y., Jiang T.Y., Jiang H.Y. (2019). Continuous Particle Trapping, Switching, and Sorting Utilizing a Combination of Dielectrophoresis and Alternating Current Electrothermal Flow. Anal. Chem..

[38] Green N.G., Ramos A., Gonzalez A., Castellanos A., Morgan H. (2001). Electrothermally induced fluid flow on microelectrodes. J. Electrostat..

[39] Sun H.Z., Ren Y.K., Tao Y., Liu W.Y., Jiang T.Y., Jiang H.Y. (2020). Combined alternating current electrothermal and dielectrophoresis-induced tunable patterning to actuate on-chip microreactions and switching at a floating electrode. Sens. Actuators B Chem..

[40] Green N.G., Ramos A., Morgan H. (2002). Numerical solution of the dielectrophoretic and travelling wave forces for interdigitated electrode arrays using the finite element method. J. Electrostat..

[41] Panklang N., Techaumnat B., Wisitsoraat A. (2020). Analysis of the equivalent dipole moment of red blood cell by using the boundary element method. Eng. Anal. Bound. Elem..

[42] Yildizhan Y., Erdem N., Islam M., Martinez-Duarte R., Elitas M. (2017). Dielectrophoretic Separation of Live and Dead Monocytes Using 3D Carbon-Electrodes. Sensors.

[43] Voldman J. (2006). Electrical forces for microscale cell manipulation. Annu. Rev. Biomed. Eng..

[44] Towhidi L., Kotnik T., Pucihar G., Firoozabadi S.M.P., Mozdarani H., Miklavcic D. (2008). Variability of the Minimal Transmembrane Voltage Resulting in Detectable Membrane Electroporation. Electromagn. Biol. Med..

